# BugMat and FindNeighbour: command line and server applications for investigating bacterial relatedness

**DOI:** 10.1186/s12859-017-1907-2

**Published:** 2017-11-13

**Authors:** Oriol Mazariegos-Canellas, Trien Do, Tim Peto, David W. Eyre, Anthony Underwood, Derrick Crook, David H. Wyllie

**Affiliations:** 1 Nuffield Department of Medicine, John Radcliffe Hospital, Headley Way, Oxford, OX3 9DU UK; 2 Public Health England Academic Collaborating Centre, John Radcliffe Hospital, Headley Way, Oxford, OX3 9DU UK; 3 Public Health England, 61 Colindale Avenue, London, NW9 5EQ UK

**Keywords:** Bacterial genomes, Phylogenetic analysis, Distance matrix

## Abstract

**Background:**

Large scale bacterial sequencing has made the determination of genetic relationships within large sequence collections of bacterial genomes derived from the same microbial species an increasingly common task. Solutions to the problem have application to public health (for example, in the detection of possible disease transmission), and as part of divide-and-conquer strategies selecting groups of similar isolates for computationally intensive methods of phylogenetic inference using (for example) maximal likelihood methods. However, the generation and maintenance of distance matrices is computationally intensive, and rapid methods of doing so are needed to allow translation of microbial genomics into public health actions.

**Results:**

We developed, tested and deployed three solutions. BugMat is a fast C++ application which generates one-off in-memory distance matrices. FindNeighbour and FindNeighbour2 are server-side applications which build, maintain, and persist either complete (for FindNeighbour) or sparse (for FindNeighbour2) distance matrices given a set of sequences. FindNeighbour and BugMat use a variation model to accelerate computation, while FindNeighbour2 uses reference-based compression. Performance metrics show scalability into tens of thousands of sequences, with options for scaling further.

**Conclusion:**

Three applications, each with distinct strengths and weaknesses, are available for distance-matrix based analysis of large bacterial collections. Deployed as part of the Public Health England solution for *M. tuberculosis* genomic processing, they will have wide applicability.

**Electronic supplementary material:**

The online version of this article (10.1186/s12859-017-1907-2) contains supplementary material, which is available to authorized users.

## Background

Whole genomic sequencing of bacteria is becoming very common, generating large sequence collections [1]. There is a both a public health and scientific need for tools which allow an understanding of the evolutionary relationships between these isolates. Bacteria typically have genomes of a few megabases; for example, *Mycobacterium tuberculosis* has a genome size of about 4.4 × 10^6^ bases [1]. Genome analysis has the potential to radically improve the detection of *M. tuberculosis* transmission [2] which is a national and global priority [3]. This is also true of multiple other organisms [4–7]. Therefore, there is a need to address the computational challenges associated with rapid detection of close relationships between sequences of the same bacterial species, including *M. tuberculosis* and other pathogenic bacterial species.

Quantification of core-genome single nucleotide polymorphism (SNP) analysis is a common initial step in relatedness estimation [2]. This step involves comparison of sequencing reads mapped to each individual base, and identifying a consensus base at each position. Filters are applied designed to exclude variation of technical origin (e.g. due to sequencing error or mismapping), marking such bases as uncertain (‘N’), and noting high-confidence consensus base calls as one of A, C, T, or G [2].

In some cases, quantification of core-genome single nucleotide polymorphisms is sufficient to generate epidemiologically important information [2]; in others, the technique is successful at identifying similar samples (samples which are very recently diverged from each other), but evolutionary inference as to the history of deep branches may be incorrect due to missing data or to recombination, and methods using maximal likelihood (ML) with or without adjustment for recombination to obtain to adjust estimates of divergence are required, e.g. [7–9]. Such methods have high computational complexity making computation slow or unfeasible for very large sequence sets, limiting their use in near-real time estimation of relatedness [8, 9]. One solution to this problem, yielding information suitable for public health purposes, involves focusing computationally complex methods on much smaller sets of similar isolates, as identified by core genome SNP estimation. Here, we discuss requirements and strategies for identifying such small sets of similar isolates.

In microbial genomic analyses, a large number of similar bacterial genomes undergo a common processing pathway independent of each other, frequently involving reference mapping in some form of high-performance computing environment. Specimens leave the environment individually, so if a computational service is provided to receive the output from such mapping, the service must support *single specimen* addition. It must additionally be capable of *rapid sequence addition*: adding individual results in an atomic, consistent, isolated and durable manner via a transaction of short duration. This is particularly important if the clients adding bacterial sequences to the relatedness service use synchronous connections to the relatedness monitoring service. Finally, the relatedness service must be *rapidly queryable*, so that computational processes consuming relatedness information can deliver closely related samples to interactive applications.

Here, we present three applications we have developed to meet these requirements. BugMat is a fast, scalable C++ application for building large distance matrices. FindNeighbour is a server based tool for maintaining, persisting and searching complete matrices, built on top of BugMat. FindNeighbour2 uses reference-based compression [10] and a database to implement rapid sparse matrix maintenance. We illustrate the use of these tools with real data from large-scale *M. tuberculosis*, *N. gonorrhoea* and *Salmonella enterica* sequencing projects. We propose that our tools will have wide application in the growing field of genomic public health microbiology, and more generally for the analysis of large sequence sets.

## Implementation

### Study of mapped data

Our applications are designed to operate on mapped data, where at each base a single base call is present. Thus, the relationship between two samples *x* and *y* can be represented using the Hamming distance:

$$ \mathit{\mathsf{h}}\left(\mathit{\mathsf{x}},\mathit{\mathsf{y}}\right)=\sum \limits_{\mathit{\mathsf{i}}=\mathsf{1}}^{\mathit{\mathsf{N}}}\mathit{\mathsf{distance}}\left({\mathit{\mathsf{x}}}_{\mathit{\mathsf{i}}},{\mathit{\mathsf{y}}}_{\mathit{\mathsf{i}}}\right),\mathit{\mathsf{i}}\;\mathit{\mathsf{\epsilon}}\;\mathit{\mathsf{sites}} $$

$$ \mathit{\mathsf{distance}}\left({\mathit{\mathsf{x}}}_{\mathit{\mathsf{i}}},{\mathit{\mathsf{y}}}_{\mathit{\mathsf{i}}}\right)=\left\{\begin{array}{c}\mathsf{0},{\mathit{\mathsf{x}}}_{\mathit{\mathsf{i}}}={\mathit{\mathsf{y}}}_{\mathit{\mathsf{i}}}\\ {}\mathsf{1},\mathit{\mathsf{otherwise}}\;\end{array}\right. $$

where *site*s are the *N* sites in *x* and *y*.

If we had precomputed *variant_sites*, where.

*variant_sites ϵ sites.*

and represents those sites which differ between *x* and *y*, then we would obtain the same distance by only considering *variant_sites*, rather than all *sites*, since the invariant sites do not contribute to the Hamming distance.

We consider the problem as one requiring construction of matrix of pairwise distances D from a set of samples X_t_ containing *t* elements x_1_ .. x_t_, such that the element D_i, j_ = h(x_i_, y_i_). We denote such a matrix as D(X_t_): for *t* = 3,

$$ D\left({X}_t\right)={\displaystyle \begin{array}{ccc}0& h\left({x}_1,{x}_2\right)& h\left({x}_1,{x}_3\right)\\ {}h\left({x}_2,{x}_1\right)& 0& h\left({x}_2,{x}_3\right)\\ {}h\left({x}_3,{x}_1\right)& h\left({x}_3,{x}_2\right)& 0\end{array}}, $$

The diagonal is zero and the matrix is symmetrical, as *h*(x_1_, *x*_2_) =  = *h*(x_2_, *x*_1_), so only half needs to be stored:

$$ \mathit{\mathsf{D}}\left({\mathit{\mathsf{X}}}_{\mathit{\mathsf{t}}}\right)={\displaystyle \begin{array}{ccc}\mathsf{0}& & \\ {}\mathit{\mathsf{h}}\left({\mathit{\mathsf{x}}}_{\mathsf{2}},{\mathit{\mathsf{x}}}_{\mathsf{1}}\right)& \mathsf{0}& \\ {}\mathit{\mathsf{h}}\left({\mathit{\mathsf{x}}}_{\mathsf{3}},{\mathit{\mathsf{x}}}_{\mathsf{1}}\right)& \mathit{\mathsf{h}}\left({\mathit{\mathsf{x}}}_{\mathsf{3}},{\mathit{\mathsf{x}}}_{\mathsf{2}}\right)& \mathsf{0}\end{array}} $$

To construct this ab initio, the asymptotic matrix construction time is $O\left(\frac{t\left(t-1\right)}{2}\right)$. For large t, this (and memory requirements) approximates $O\left({\frac{t}{2}}^2\right)$.

### Iterative addition of samples

In the use case we are considering, we do not know the entire set of X_t_ samples when we start matrix construction, since samples are provided individually. Rather, we are required to iteratively add samples, progressively increasing matrix size in a series of t steps, where t is 1, 2, 3 and so on.

Thence, to add a fourth sample to the matrix above, we are required to compute (t-1) matrix entries

$$ \mathit{\mathsf{D}}\left({\mathit{\mathsf{X}}}_{\mathit{\mathsf{t}}}\right)={\displaystyle \begin{array}{cccc}\mathsf{0}& & & \\ {}\mathit{\mathsf{h}}\left({\mathit{\mathsf{x}}}_{\mathsf{2}},{\mathit{\mathsf{x}}}_{\mathsf{1}}\right)& \mathsf{0}& & \\ {}\mathit{\mathsf{h}}\left({\mathit{\mathsf{x}}}_{\mathsf{3}},{\mathit{\mathsf{x}}}_{\mathsf{1}}\right)& \mathit{\mathsf{h}}\left({\mathit{\mathsf{x}}}_{\mathsf{3}},{\mathit{\mathsf{x}}}_{\mathsf{2}}\right)& \mathsf{0}& \\ {}\mathit{\mathsf{h}}\left({\mathit{\mathsf{x}}}_{\mathsf{4}},{\mathit{\mathsf{x}}}_{\mathsf{1}}\right)& \mathit{\mathsf{h}}\left({\mathit{\mathsf{x}}}_{\mathsf{4}},{\mathit{\mathsf{x}}}_{\mathsf{2}}\right)& \mathit{\mathsf{h}}\left({\mathit{\mathsf{x}}}_{\mathsf{4}},{\mathit{\mathsf{x}}}_{\mathsf{3}}\right)& \mathsf{0}\end{array}} $$

Addition of a single sample thus scales linearly with t, taking O(t-1) time. Nevertheless, a large number of sequence positions still need to be analysed.

### Optimisation of pairwise hamming distance computation using a variation model

A potential optimisation concerns the computation of pairwise Hamming distances between samples. Bacterial genomes are typically about 5 × 10^6^ nucleotides; the *M. tuberculosis* reference strain using for mapping in this work is 4.4 × 10^6^ nucleotides. In common with many other bacteria, *M. tuberculosis* has a strong population structure, and comprises multiple ancient lineages which differ from each other by about 2000 ancient SNPs [11]. Thus, if we wish to compute difference matrices between *M. tuberculosis* genomes, the number of variant positions is expected to be much smaller than the genome size, perhaps a few thousand SNPs only.

If we know that there is no variation at position *i* between the *t* sequences to be analysed, there is no need to perform pairwise comparisons between all *t* samples at this site, a process with ~O(*t* ^2^) complexity for large *t*. Rather, we can assign a Hamming distance of 0 for all invariant sites without pairwise evaluation. To allow this, we include code to maintain a vector of variant sites in the alignment, which we term the *variation model*. We maintain the *variation model* in a datastructure containing counts of all bases at each position. This model, which allows rapid assessment of whether sites are variant is updated on addition of each sample if and only if the position is not currently variant, since positions cannot become invariant as further samples are added. Pseudocode for this operation is in Additional file 1.

The asymptotic time for addition of a single sample to the variation model is O(1), and allows large (~ 1000 ×) speedup in the subsequent expensive pairwise distance computation since most sites are invariant. Storage of the matrix allows addition of samples to a collection of t samples with a cost of O(*t*-1), rather than ~ O(*t* ^2^) for complete matrix recomputation.

### Reference based compression

There is an alternative formulation of the problem. In this, the mapped sequence S is represented a series of sets S_A_, S_C_, S_G_, S_T_ containing positions of non-reference A, C, G and T bases, and S_N_ containing the positions of uncallable bases. This is an implementation of reference based compression [10], but importantly one in which the compression strategy aids in the computation of the Hamming distance *d* between two sequences R and S using set operations:

$$ d=\sum \limits_{b\in \left\{A,C,T,G\right\}}\left|\right.\left({S}_b-{R}_N\right)\varDelta \left({R}_b-{S}_N\right)\left.\right| $$

Since Python has an interface to highly optimised C++ set operations, we elected to code the implementation in Python 3.

### Presentation

We present two solutions using the matrix-based approach, and one using the set-based approach. **BugMat** is a C++ command line executable, tested on both Linux (using gcc) and Windows (using MinGW), which performs rapid one-off distance matrix construction using a two phase computation, firstly building an index of variant bases in the sequences, and subsequently performing multi-threaded computation of pairwise distances between every sample pair with shared memory to store all data.

The second is **FindNeighbour**, a server application, built with the python web.py framework, and accessed using a XMLRPC API. Internally, it comprises two components. Firstly, an OpenMP parallelised C++ application derived from BugMat maintains an in-memory distance matrix derived from mapped genomic data. Secondly, a database allows storage and querying of arbitrary meta-data about the sequence. FindNeighbour uses this database internally to store quality information about the sequence, such as the number of bases called in the sequence (i.e. bases called as A, C, T, G), and timing information related to server activity. This is at present implemented using an on-disc relational database via an object relational mapper. Additionally, FindNeighbour performs automated matrix persistence to disk, and recovers stored matrices from disk on server restarts.

Design of BugMat/Findneighbour software includes a series of technical optimisations supporting the implementation of the variation model described above. Firstly, we have chosen to code to core of our software in C++, which can be compiled using gcc with the O3 and funroll-loops optimisations flags. We have elected to minimise disc access overhead by reading sequences into shared memory, which imposes a memory cost but allows fast, dynamic read/write using the unordered_map data type, which is faster than the C++ standard library’s map type. Finally, we have parallelised pairwise comparisons using the openmp library, based on a fork-join paradigm.

The third application is **FindNeighbour2.** This compresses the sequences supplied to it, and stores them both in RAM and on disc following reference-based compression. On addition of a sample, it is compared with all other samples and matches below a customisable threshold are written to a database. Otherwise, the implementation is similar to FindNeighbour.

For both applications, identical API methods allow:

- addition of samples, comprising an identifier and a fasta sequence;
- testing whether an identifier is present;
- determining distance between a pair of samples;
- determining neighbours of one sample or all samples, given a sequence quality threshold.

Table 1 summarises the similarities and differences between these technologies.

**Table 1:** Comparison of three solutions

Implementation	BugMat	FindNeighbour1	FindNeighbour2
Presentation	Command Line	Server application	Server application
Technology	In-memory matrix	In-memory matrix	In-memory reference based sequences
Can add samples	No	Yes	Yes
Role in production environment	Remove invariant sites before maximal likelihood tree generation	Store pairwise distances between samples	Store significant pairwise distances between samples
Stores all pairwise distances	Yes	Yes	No (customisable)
Uses database for sequence metadata storage	No	Yes	Yes
Uses database for pairwise distance storage	No	No	Yes
Requires reference sequence specified	No	No	Yes
Implementation	C++	Python, C++	Python

Comparison of three solutions. A comparison of the approaches taken by three solutions (BugMat, Findneighbour, Findneighbour2) is shown

## Results and discussion

We initially tested the performance of both the BugMat and FindNeighbour tools using real data comprising mapped data from bacterial sequencing projects.

### Matrix based methods

**Fig. 1:**
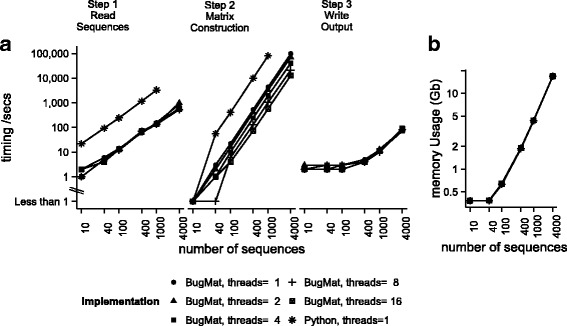
Performance of the BugMat application. The relationship between number of sequences processed, numbers of cores assigned to BugMat, and (**a**) memory usage and (**b**) various stages of sequence processing. Testing was performed a Ubuntu 14 VM instance with 16 cores, Intel Xeon E5-2680v2 processors at 2.8GHz, and 128GB RAM hosted within Genome England Ltd. Similar results were obtained using the specification in Additional file 2

We tested the command-line **BugMat** executable using data set A (*M. tuberculosis,* Table 2). The initial *variant model* construction step is computationally inexpensive (Fig. 1), and allows subsequent fast pairwise distance matrix construction using only variant positions. With more than 100 sequences, time for matrix construction dominates runtime; with fewer samples, about half the runtime involves reading and writing file output (Fig. 1). With 100 samples, BugMat computed a distance matrix in fewer than 4 s using 16 cores. Four hundred samples took 70 s, and 4000 sequences took about 3 h seconds, with the latter requiring 20GB of RAM. This tool forms part of a production pipeline, and is used to strip out invariant sites prior to maximal likelihood tree generation for up to 500 related isolates.

**Table 2:** Data sets used for testing findNeighbour performance

Dataset	Sites called	Links less than SNP stored	Memory usage	Mean time to add one sample
A: *M. tuberculosis*, *n* = 15,985 mapped to NC_000962	329,714 sites excluded	20	23.5G	2.23 s
B: *Neisseria gonorrhoea*, *n* = 2455 mapped to NC_011035	All sites included	500	19.3G	2.95 s
C: *Salmonella enterica*, *n* = 5380 mapped to AM933172	51,897 sites excluded	20	7.4G	1.77 s

Data sets studied and FindNeighbour2 performance

The data sets studied, which can be downloaded at https://ora.ox.ac.uk/objects/uuid:82ce6500-fa71-496a-8ba5-ba822b6cbb50 are described. Also shown are performance characteristics of Findneighbour2 operating on them using the hardware in Additional file 2

BugMat has various restrictions. Firstly, unlike sophisticated software capable of performing large-scale sequence alignment and distance matrix generation [12], it relies on pre-aligned sequences. It splits the workload across cores, but does not support distribution of work across multiple computational nodes [12]. Secondly, it is presently restricted to CPU-based architectures supporting OpenMP, including the emerging Intel Xeon Phi [13] architecture; however, similar sharding of tasks across multiple computational cores can be achieved using graphical processing units and CUDA. Thirdly, by design it builds matrices in memory. This imposes limits on matrix size which could be avoided introducing a disc-based storage capability, albeit at large performance cost. Finally, it builds matrices ab initio; it does not allow their updating, and has high rebuild times for large sample numbers.

**Fig. 2:**
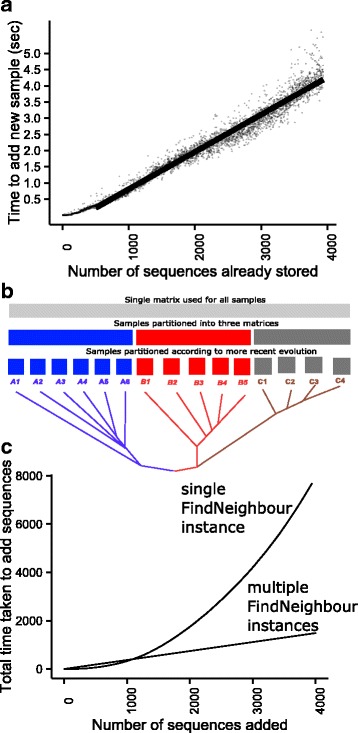
Performance of the FindNeighbour application. The time taken to add 4000 individual *M. tuberculosis* samples to a FindNeighbour server (Panel **a**) Testing was performed a Ubuntu 14 VM instance with 16 cores, Intel Xeon E5-2680v2 processors at 2.8GHz, and 128GB RAM hosted within Genome England Ltd. Samples which diverged from each other at various times in the past (depicted in Panel **b**) can either be added to one FindNeighbour instance, or multiple (63, see text) instances. The performance of these strategies is illustrated in Panel **c**

This last limitation is restrictive for public health applications in which ongoing comparison of isolates with a databank is required. **FindNeighbour** addresses this. Using the same test set as for BugMat, the time to add new samples to FindNeighbour is directly proportional to the number of previous samples present, as is expected from the internal software architecture (Fig. 2a). Server response times recovering neighbours within 20 single nucleotide variations of a sequence averaged 50 msec with 4000 samples stored. The application is very stable, and a production instance with over 21,500 samples on a 128GB RAM server (110GB RAM used) is operational as part of a Public Health England initiative to sequence all TB in England. Importantly, however, as with BugMat, memory requirements increase quadratically, and this limits further scalability.

One approach to this is to deploy multiple FindNeighbour instances as part of a map-reduce paradigm for the analysis of very large numbers of sequences; the *map* step depends on sequence features (Fig. 2b). Each of groups derived from the *map* operation can be processed by independent FindNeighbour instances, perhaps deployed on the same server. As an example, we have shown that a published method of subdividing *M. tuberculosis* based on ancestry [14] into 63 subgroups, can be readily accommodated with about 4× speed-up in the storage of 4000 samples and marked reductions in the amount of memory required (Fig. 2c). This *map* process will precede reduction, in which the responses of the relevant FindNeighbour instance are returned to the client. However, quadratic memory requirements will still limit scalability.

A second approach, which is not necessarily mutually exclusive, is to move away from technologies which have quadratic memory requirements. **FindNeighbour2** implements such, using reference based compression of mapped sequence data, producing linear memory requirements with rising sequence numbers. In Table 2, we summarise the performance of this technology on a series of test datasets, with various different clinically relevant cutoffs.

Enhancements to FindNeighbour2 are planned. A prototype randomly assigns sequences to a series of FindNeighbour2 instances, each running with a single thread; one instance stores each new sequence. Organism-specific algorithms assigning an organism ‘to the right compartment’ for analysis are not attempted, as these require validation; rather, a new-against-all-existing comparison is performed, sharded across the instances. This has the advantage that true matches are guaranteed to be found, something which may be essential, in view of the clinical importance of identification of close neighbours for initiating public health action. However, as numbers of samples grow, the requirement to perform a new-against-all comparison may become rate limiting. If it does so, then approximate nearest neighbour matching could be deployed, using each variant locus as a dimension in a multi-dimensional clustering algorithm; algorithms exist which allow highly efficient selective searching of such data using k-d forests or k-means search trees [15].

In 2015, there were about 3 million cases of TB confirmed by culture globally [16]. Even if only 5% of these are sequenced annually, the generation of 1 M bacterial sequences will be achieved in the next decade. With a comparison rate of 15,000 sequences/s/core, and an in-memory footprint of about 2 MB/sequence, we project that we could store these in about 2 TB of RAM, either on a single server, or in a distributed manner. If 80 computational cores were available, the time to search for neighbours among these would approximate 1–2 s. The commercial cost of a single server with suitable hardware is at present about GBP 50,000, making the approach we describe using reference based compression a candidate for tracking of TB relatedness in large, international datasets, if reference based mapping approaches are used.

## Conclusions

The FindNeighbour tools are, to our knowledge, first-in-class applications allowing progressive addition of mapped sequence data to a sequence store, thence allowing rapid identification of neighbours based on SNP distance data derived from reference mapping. These tools make no assumptions about organism phylogeny in their operation. Therefore, we believe these tools will have applicability to a range of applications in bacterial genomics.

## Availability and requirements

For project home pages, please see section below. Operating systems and languages used are shown inTable. The software is released under LGPL licence.

## Additional files

Additional file 1: Pseudocode for maintaining a variation model. (PDF 271 kb)

Additional file 2: Environment used for FindNeighbour and Bugmat performance testing. (PDF 272 kb)

## References

[1] Didelot X, Bowden R, Wilson DJ, Peto TE, Crook DW (2012). Transforming clinical microbiology with bacterial genome sequencing. Nat Rev Genet.

[2] Walker TM, Monk P, Smith EG, Peto TE (2013). Contact investigations for outbreaks of mycobacterium tuberculosis: advances through whole genome sequencing. Clin Microbiol Infect.

[3] Collaborative Tuberculosis Strategy for England 2015 to 2020. Available at: https://www.gov.uk/government/publications/collaborative-tuberculosis-strategy-for-england. Accessed 8 Nov 2017. 10.1136/bmj.h810 25697392

[4] Inns T, Ashton PM, Herrera-Leon S, Lighthill J, Foulkes S, Jombart T, Rehman Y, Fox A, Dallman T, DEP E (2017). Prospective use of whole genome sequencing (WGS) detected a multi-country outbreak of salmonella Enteritidis. Epidemiol Infect.

[5] Gordon NC, Pichon B, Golubchik T, Wilson DJ, Paul J, Blanc DS, Cole K, Collins J, Cortes N, Cubbon M, et al. Whole genome sequencing reveals the contribution of long-term carriers in Staphylococcus Aureus outbreak investigation. J Clin Microbiol. 2017; 10.1128/JCM.00363-17 PMC5483921 28468851

[6] De Silva D, Peters J, Cole K, Cole MJ, Cresswell F, Dean G, Dave J, Thomas DR, Foster K, Waldram A (2016). Whole-genome sequencing to determine transmission of Neisseria gonorrhoeae: an observational study. Lancet Infect Dis.

[7] Eyre DW, Cule ML, Wilson DJ, Griffiths D, Vaughan A, O'Connor L, Ip CL, Golubchik T, Batty EM, Finney JM (2013). Diverse sources of C. Difficile infection identified on whole-genome sequencing. N Engl J Med.

[8] Nguyen LT, Schmidt HA, von Haeseler A, Minh BQ (2015). IQ-TREE: a fast and effective stochastic algorithm for estimating maximum-likelihood phylogenies. Mol Biol Evol.

[9] Didelot X, Wilson DJ (2015). ClonalFrameML: efficient inference of recombination in whole bacterial genomes. PLoS Comput Biol.

[10] Numanagic I, Bonfield JK, Hach F, Voges J, Ostermann J, Alberti C, Mattavelli M, Sahinalp SC (2016). Comparison of high-throughput sequencing data compression tools. Nat Methods.

[11] Comas I, Coscolla M, Luo T, Borrell S, Holt KE, Kato-Maeda M, Parkhill J, Malla B, Berg S, Thwaites G (2013). Out-of-Africa migration and Neolithic coexpansion of mycobacterium tuberculosis with modern humans. Nat Genet.

[12] Al-Neama MW, Reda NM, Ghaleb FF (2014). An improved distance matrix computation algorithm for multicore clusters. Biomed Res Int.

[13] Misra S, Pamnany K, Aluru S (2015). Parallel mutual information based construction of genome-scale networks on the Intel(R) Xeon phi coprocessor. IEEE/ACM Trans Comput Biol Bioinform.

[14] Coll F, McNerney R, Guerra-Assuncao JA, Glynn JR, Perdigao J, Viveiros M, Portugal I, Pain A, Martin N, Clark TG (2014). A robust SNP barcode for typing mycobacterium tuberculosis complex strains. Nat Commun.

[15] Muja M, Lowe DG (2014). Scalable nearest neighbor algorithms for high dimensional data. IEEE Trans Pattern Anal Mach Intell.

[16] Vijay S, Dalela G (2016). Prevalence of LRTI in patients presenting with productive cough and their antibiotic resistance pattern. Journal of clinical and diagnostic research : JCDR.

