# Supplementary material for: BugMat and FindNeighbour: command line and server applications for investigating bacterial relatedness
Source: BMC Bioinformatics. 2017 Nov 13;18:477. doi: 10.1186/s12859-017-1907-2 (PMC5683244; doi:10.1186/s12859-017-1907-2)
Supplement: Supplementary file 1 — Pseudocode for maintaining a variation model. (PDF 271 kb) [file 12859_2017_1907_MOESM1_ESM.pdf]

## Additional file 1: Pseudocode for assessing variation

We start with a t samples comprising mapped data, a set of samples we call X.

Each sample in X is of length L. The objective is to maintain a vector variant_sites of length L containing either the invariant base present at each position (one of A,C,G,T) or a character ('.') indicating variation. On addition of the $t^{th}$ sample $X_{t:}$

Pseudocode:

```
bases = [A,C,T,G]

alphabet = [N]

for i := 1 to L do

        if variant_sites[i] is not '.' and Xt[i] in bases and variant_sites[i] != Xt[i] then

                variant_sites[i] = '.'

end
```
