# Supplementary material for: BugMat and FindNeighbour: command line and server applications for investigating bacterial relatedness
Source: BMC Bioinformatics. 2017 Nov 13;18:477. doi: 10.1186/s12859-017-1907-2 (PMC5683244; doi:10.1186/s12859-017-1907-2)
Supplement: Supplementary file 2 — Environment used for FindNeighbour and Bugmat performance testing. (PDF 272 kb) [file 12859_2017_1907_MOESM2_ESM.pdf]

Additional file 2: Environment used for FindNeighbour and Bugmat performance testing

Property	Result
Server	Baremetal server running Ubuntu 16.04 LTS
RAM	128G RAM DDR3
Cpu	2 x Intel Xeon 2.4GHz
Cores used	1 for server, 1 for database
Database	Postgres on localhost.
Database disc	Same as storage disc
Storage disc	Storage of fasta and reference compressed data on 12 TB RAID5 device (4 x 4TB discs, SATA, 7200 rpm)
